# Drotaverine to shorten the duration of labour in primigravidas: a randomised, double-blind, placebo-controlled trial

**DOI:** 10.4314/ahs.v22i3.13

**Published:** 2022-09

**Authors:** Arinze Chidiebele Ikeotuonye, Odidika Johannes Umeora, Johnbosco Ifunanya Nwafor, Bonaventure Omuye Ojumah, Ifeoma Cecilia Ekwunife, Ikechukwu Bonaventure Dimejesi

**Affiliations:** Department of Obstetrics and Gynaecology, Alex Ekwueme Federal University Teaching Hospital, Abakaliki, South-east, Nigeria

**Keywords:** drotaverine, duration of active phase of labour, primigravidas, placebo, shortening

## Abstract

**Background:**

Drotaverine, a spasmolytic, has been found to have potential to achieve a reduction in the duration of labour and prevent prolonged labour.

**Objective:**

To compare the effects of intravenous drotaverine hydrochloride with placebo for shortening the duration of active phase of labour in primigravidas.

**Methods:**

A double-blind, placebo-controlled randomized trial of 246 primigravidas in active phase of labour at term was conducted. They were randomly (1:1 ratio) administered intravenous 2 ml (40mg) of drotaverine hydrochloride or 2 ml of Vitamin B complex as placebo. The primary outcome measure was the duration of active phase of labour. The secondary outcome measures were cervical dilatation rate, oxytocin augmentation rate, incidence of prolonged labour, labour pain scores, mode of delivery, maternal and neonatal outcomes.

**Results:**

The mean duration of active phase of labour (hour) was significantly lower in the drotaverine group compared to the control (drotaverine; 6.22 ± 2.41 vs placebo; 8.33 ± 3.56; p <0.001). Also, the cervical dilatation rate (cm/hr) was significantly faster in the drotaverine arm (drotaverine; 1.68 ± 1.02 versus placebo; 1.06 ± 0.53, p <0.001). There was a significantly higher probability of faster delivery among women who were given drotaverine (log-rank test, p < 0.001). The oxytocin augmentation rate, incidence of prolonged labour, labour pain scores, mode of delivery, maternal and neonatal outcomes were not significantly different among the groups.

**Conclusions:**

Drotaverine hydrochloride is effective in shortening the duration of active phase of labour without adverse maternal and neonatal outcomes. However, more evidence is needed to explore its role in active phase of labour among primigravid women. Trial registration number: PACTR201810902005232.

## Introduction

Prolonged labour is associated with increased maternal and perinatal complications such as fetal distress, birth asphyxia, neonatal admission into the intensive care unit, postpartum haemorrhage and sepsis which are major contributors to adverse pregnancy outcome.^1^ Therefore, minimizing the duration of labour without compromising the fetal or maternal outcome is important, especially for the primigravidas who are at higher risk of prolonged labour and its associated complications.^1^

The risk for complications of prolonged labour is even more pronounced for resource poor settings, as we are often less prepared logistically to handle the maternal or fetal complications that may be associated with it. Reduction of the duration of labour even becomes more important in cases where there is uteroplacental insufficiency encountered in conditions such as hypertensive disorders of pregnancy and women with diabetes mellitus in pregnancy when vaginal birth is not contraindicated.^2^ The major factors that determine the progress of labour include cervical dilation, descent of presenting part and uterine contractions.^3^ Various attempts have been made towards a reduction in the duration of labour through the modulation of these important factors that determine the progress of labour. Oxytocin, prostaglandins and antispasmodics are some of the broad classes of pharmacological agents that have been used in the modification of these factors.^2^ Membrane sweeping and cervical stretching are other mechanical methods that have been used with varying degrees of success and these methods apply because of the role of the cervix in the labour process.^4^,^5^ The cervix undergoes softening and dilatation which contributes to the progress of labour. Cervical softening and dilatation can be influenced by uterine contractions, mechanical and pharmacological agents.^5^

Antispasmodic drugs are acceptable agents which can hasten cervical dilatation during labour.^2^ The actions of antispasmodics could be through neurotropic and/or musculotropic effects.^6^ Drotaverine hydrochloride is a musculotropic antispasmodic agent and an inhibitor of type IV phosphodiesterase, structurally related to papaverine.^7^ It also has mild calcium channel blocking activity but no anticholinergic effects unlike hyoscine butyl bromide.^7^ It acts directly on the smooth muscle cells inhibiting spasm.^7^

The contribution of cervical spasm to delay in cervical dilatation and the exact mechanism through which antispasmodics, including drotaverine hydrochloride may hasten cervical dilatation has not been fully elucidated.^8^ There is however an increasing pool of evidence suggesting a significant reduction in the duration of the first stage of labour and an increase in cervical dilatation rate for patients who received drotaverine hydrochloride in labour.^2^,^8^ Although the World Health Organisation (WHO) published a weak recommendation based on very low-quality evidence against giving antispasmodics to reduce the duration of labour as they did not consider a 1-hour reduction clinically significant especially with limited safety data, the WHO guideline development group considered studying the use of antispasmodics to treat labour delay a research priority.^9^ Apresent, few studies have evaluated the role of drotaverine in labour management in the background of active management of labour or a standard intrapartum protocol. Therefore, the aim of this study was to determine the effects of drotaverine in shortening the duration of active phase of labour in primigravidas with term pregnancy.

## Methods

### Study design and setting

This is a superiority, double-blind, randomised controlled clinical trial conducted from 1^st^ May, 2018 to 31^st^ October, 2018 at Alex Ekwueme Federal University Teaching Hospital, Abakaliki and its affiliate, Mile 4 maternity hospital, Abakaliki.

Alex Ekwueme Federal University Teaching hospital (formerly known as Federal Teaching Hospital), Abakaliki caters for both primary and referral cases from Ebonyi state and environs including Enugu, Cross river, Abia and Benue states and other parts of the country.10 The consultants and the resident doctors, nurses and other ancillary health workers run the clinics. Each unit in the department of Obstetrics and Gynaecology is manned by a group of Consultants with senior Registrars, Registrars and House Officers.

Mile 4 hospital is a missionary hospital where resident doctors from the Obstetrics and Gynaecology department of the Alex Ekwueme Federal University Teaching Hospital Abakaliki rotate for labour ward and rural postings in Obstetrics and Gynaecology. It is about 2 miles away from the Alex Ekwueme Federal University Teaching hospital, Abakaliki. The Obstetrics and Gynaecology Department of Mile 4 hospital is run by two consultants, senior registrars and residents posted from the Obstetrics and Gynaecology department of the Alex Ekwueme Federal University Teaching Hospital, Abakaliki.

### Study participants

This study was conducted among primigravidas with term pregnancy admitted in active phase of labour in the study facilities during the study period.

The eligible participants were consenting primigravid women at term (gestational age 37+0 - 41+6 weeks) in active phase of labour carrying singleton foetus in cephalic presentation and cervical dilatation of 4 - 5 cm. The exclusion criteria for this study were the presence of either one or more of the following: abnormal fetal presentation, non-reassuring fetal status, multiple gestation, antepartum haemorrhage, coexisting uterine fibroid, previous uterine or cervical surgery, medical disorders in pregnancy or refusal to give consent.

On admission, the gestational age was calculated according to the Naegele rule and confirmed by reviewing early pregnancy ultrasound scans. Active labour was diagnosed by the presence of regular uterine contractions (each lasting for 30 seconds or more) at a rate of at least 2 every 10 minutes, with or without rupture of membranes and cervical dilatation of 4 – 5 cm.

### Enrollment and Randomisation

Women attending prenatal care at the study facilities were informed about the study and asked to consider participation before their admission to the hospital with signs and symptoms of possible labour onset. Women attending the labour ward/labour assessment unit between 37+0 and 41+6 weeks of gestation with signs and symptoms of possible labour onset (e.g. regular uterine contractions, rupture of membranes, maternal report of suspected labour), and who had received the study information, were screened for eligibility to participate in the trial using a trial screening and register form. Screening was conducted by the admitting specialist registrar, on a 24-hour basis, as women presented. Women who were screened eligible were invited to participate in the trial and sign the study consent form.

**Figure 1:**
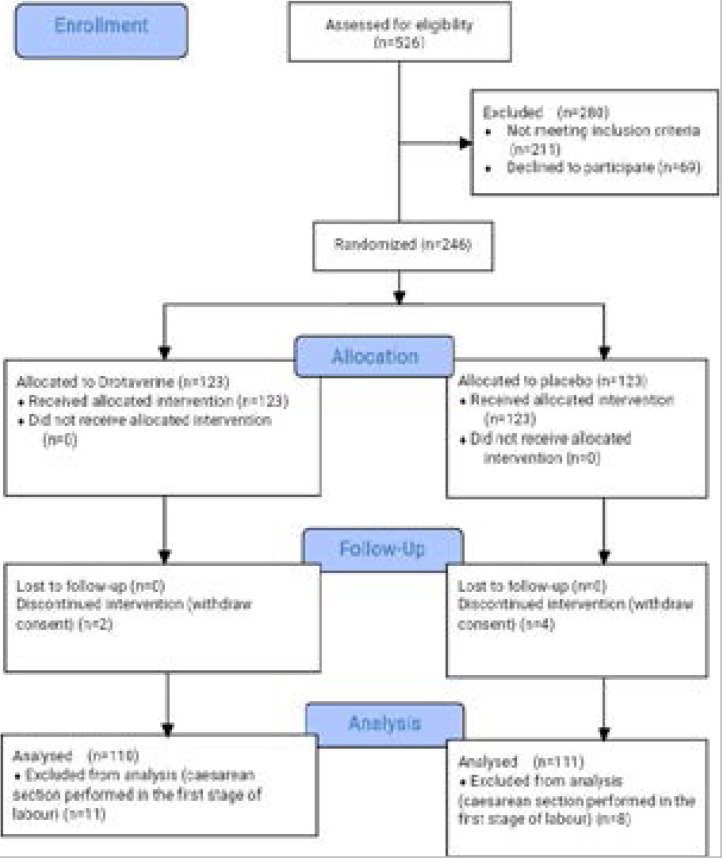
CONSORT chart showing flow of the participants through the study.

An independent statistician generated a sequence of random numbers list using the online randomisation service of www.randomizer.org in permuted block sizes of four, six and eight. The list was given to the hospital pharmacist, who prepared the drugs with defined letters A or B (drotaverine or Vitamin B complex). Standardised, sequentially numbered, identical opaque envelopes were prepared in accordance with the randomisation list. Each envelope contained either drotaverine or Vitamin B complex ampoule. Information on the content of each envelope was masked until the completion of data analysis. Participants were randomly allocated to either study Group A or B in a 1:1 ratio. The envelopes were kept in the labour ward and were serially drawn by midwives, who were not part of the study, until the study was completed. After an eligible participant had given consent, she was assign a number by the investigator who then called a midwife to draw the corresponding envelope. The envelop was given to the attending specialist registrar, who was not part of the study, to administer the drug to the participants as shown on the allocation paper in the envelope. The researchers and the participants were masked to the group allocation. Half of the participants were recruited from Alex Ekwueme Federal University Teaching Hospital, Abakaliki and the remaining half from Mile 4 hospital, Abakaliki with equal mixes of the drug and placebo used at any time in both institutions to correspond to the required number of 111 participants per arm. The flow of the participants through the study is shown in Figure 1.

### Intervention group

The participants in the drotaverine arm received 2 ml (40 mg) drotaverine (SanofiAventisR) Pharmaceutical Company, Lagos, Nigeria). The drotaverine was administered intravenously as a bolus immediately after recruitment by trained research assistants who were specialist registrars working in the labour suite.

### Control group

The participants in the placebo arm received 2 ml of Vitamin B complex (a product of Juhel, Enugu, Nigeria), which was administered intravenously as a bolus immediately after recruitment by trained research assistants.

In addition, participants were managed according to the local institutional intrapartum protocol.^11^ The presence of normal fetal heart rate pattern was established for 30 minutes before the intervention was started. Intrapartum fetal monitoring was performed via intermittent auscultation using Pinard stethoscope while women who had augmentation of labour were monitored with cardiotocograph. Artificial rupture of fetal membranes was done routinely for women with intact membranes at randomisation as per unit protocol. Vaginal examination after randomization was performed at 4-hour and then every 2 hours, unless clinically indicated, to define the progress of cervical dilatation. For participants who had poor progress of labour (cervical dilatation rate <1 cm/hour), augmentation of labour was done using oxytocin infusion with a starting dose of 2 mU/minute and increments of 2 mU/minute every 15 minutes until adequate uterine contractions were achieved or the maximum dose (32 mU/minute) was reached. The intrapartum fetal heart rate patternnd uterine contractions were interpreted in accordance with NICE guidelines.^12^ A 4-hour action line partograph was used to document the progress of labor and the duration of the first, second and third stages of labor to determine the study endpoints. The third stage of labour was managed actively.

### Outcome measures

Although, a core outcome set for assessing studies on shortening the duration of active phase of first stage of labour is not registered with the Core Outcome Measures in Effectiveness Trials (COMET) database (http://www.cometinitiative.org), and this core outcome set is not yet developed. In the absence of a core outcome set, our outcome measures were as follows. The primary outcome measure was the duration of active phase of labour. The secondary outcome measures were cervical dilatation rate, oxytocin augmentation rate, pain scores (assessed using Visual Analogue scale [VAS] at enrollment, 30, 60 and 120 minutes after administration of intervention), incidence of prolonged labour (injection to full cervical dilatation >12 hours), Maternal drug side effects (headache and nausea), mode of delivery, estimated blood loss and neonatal outcomes such as Apgar scores at 1^st^ and 5^th^ minutes of birth and admission into neonatal intensive unit. The following baseline characteristics were also measured: maternal age, occupation, religion, marital status and area of residence. The participants were followed up to delivery and the the outcomes measures were assessed at delivery, apart from pain assessment using VAS.

### Sample size and power calculation

A priori power analysis was performed before the study for sample size estimation using G*Power version 3.1.9.2 software.^13^ We estimated an effect size of a 0.39 based on a previous study.14 To obtain a power of 80% at a 5% significance level with an effect size of 0.39, an allocation ratio N2/N1 of 1 and a two-tailed test, a minimum sample of 210 participants were required. By adding 5% attrition rate, the total sample size was 220.5. Therefore, 222 clients were recruited into the study and randomly assigned to each arm of the study.

### Data collection and statistical analysis

Data was extracted from the hard copy clinical notes in each hospital. Predesigned data extraction form was developed, piloted and refined for collecting the relevant data. Before hospital discharge, the participants filled a questionnaire containing information on the occurrence of side effects during the study period. All data were entered into Microsoft excel and imported into SPSS version 22 for analysis using per protocol principle. Analysis was also done by Intention-to-treat method (see supplementary Tables). The distribution of the continuous variables were checked using histogram with superimposed normal distribution curve. Data that showed normal distribution were analysed using parametric tests whereas those that were skewed were analysed using non-parametric tests. Descriptive statistics was used to summarise baseline characteristics of the study participants. Categorical variables were compared using the Chi-square (χ2) or Fisher's exact tests where as continuous variables were compared using independent sample ‘t’ test or Mann Whitney U test. For dichotomous data, relative risks (RR) with 95% CI was calculated. A Kaplan-Meier survival analysis was used to detect the probabilities of faster delivery with the use of drotaverine hydrochloride and placebo. The 2 curves were compared via a log rank test. A p value < 0.05 was considered statistically significant.

### Ethical consideration

Ethical approval to conduct the study was granted by the Research and Ethics Committee of the Alex Ekwueme Federal University Teaching Hospital, Abakaliki (Approval number: FETHA/REC/VOL1/2017/570). Also, the study was registered with the Pan African Clinical Trial Registry (www.pactr.org) database (identification number: PACTR201810902005232). Participation was voluntary, and participants were informed that they could withdraw from the study at any time. Written informed consent was obtained from eligible women as close as possible to the time of randomisation. All data were collected, processed and stored confidentially. The study was carried out as per the guidelines given in the Declaration of Helsinki 2013. The study was monitored by data safety committee of both hospitals so as to ensure the safety of the study participants. There was no serious adverse event during the study.

### Patient and public involvement

The patients and public were not involved in the design and conceptualisation of this study.

## Results

During the study, 526 women were assessed for eligibility to participate in the study. Of those assessed, 280 women were excluded: 211 did not meet the inclusion criteria and 69 parturients declined to participate in the trial. 246 participants who met the inclusion criteria and gave their consent to be enrolled in the study were randomly assigned to either drotaverine group (n=123) or placebo group (n=123). The flow of the participants through the study is shown in Figure 1.

In the drotaverine arm of the study, 2 participants withdrew consent to continue the study after receiving intervention and 11 participants had emergency caesarean section in the first stage of labour. The indications for the caesarean sections were intrapartum abruptio placentae (n=2), cord prolapse (n=1) and cephalopelvic disproportion (n=8). Participants who had emergency caesarean section were excluded from data analysis. Therefore, 110 participants who completed the study, were included in the data analysis.

For those allocated to the placebo arm of the study, 111 participants completed the study and they were included in the data analysis. Of the 12 participants excluded from data analysis, 4 women withdrew their consent to continue the study whereas 8 participants had emergency caesarean section in the first stage of labour. Among those who had caesarean section, 6 was due to cephalopelvic disproportion and 2 due to non reassuring fetal heart rate pattern on cardiotocograph.

The baseline characteristics of the study groups are shown in Table 1. There were no difference between the study groups as regards to age (years) (p = 0.549), body mass index (p = 0.786), gestational age at delivery (weeks) (p = 0.363) and cervical dilatation at randomization (cm) (p = 0.135).

**Table 1:** Baseline characteristics of the study participants

Variable	Drotaverine (n=110) Mean ± SD	Placebo (n=111) Mean ±SD	*p* value*
Maternal age (years)	24.1 ± 3.4	24.3 ± 3.6	0.549
BMI (kg/m^2^)	23.6 ± 3.8	24.1 ± 3.9	0.786
Gestational age (weeks)	39.4 ± 1.2	39.1 ± 1.1	0.363
Cervical dilatation (cm)	4.23 ± 1.3	4.24 ± 1.5	0.135

* Derived from independent sample “t” test

SD = standard deviation

Table 2 shows the comparison of the study outcomes between the study groups. The 2 groups differed in the rate of cervical dilatation (drotaverine; 1.68 ± 1.02 versus placebo; 1.06 ± 0.53, p <0.001) and the duration of active phase of labour (drotaverine; 4.21 ± 1.98 versus placebo; 6.61 ± 2.79, p <0.001). However, there was no significant difference between the duration of second stage of labour (drotaverine; 0.76 ± 0.37 versus placebo; 0.78 ± 0.39, p = 0.582) and the duration of third stage of labour (drotaverine; 0.16 ± 0.07 versus placebo; 0.16 ± 0.08,/span>p = 0.163) in both study arms. Overall, the total duration of labour was lower in drotaverine group when compared to the placebo group (drotaverine; 6.22 ± 2.41 versus placebo; 8.33 ± 3.56, p <0.001).

**Table 2:** Comparison of the outcomes among the study groups

Variable	Drotaverine (n=110)	Placebo (n=111)	RR (95% CI)	p value
Primary outcome measure				
Mean duration of active phase of labour (hour) ± SD	4.21 ± 1.98	6.61 ± 2.79		<0.001*
Secondary outcome measures				
Mean cervical dilatation rate (cm/hr) ± SD	1.68 ± 1.02	1.06 ± 0.53		<0.001*
Mean duration of second stage of labour (hour) ± SD	0.76 ± 0.37	0.78 ± 0.39		0.582*
Mean duration of third stage of labour (hour) ± SD	0.16 ± 0.07	0.16 ± 0.08		0.163*
Mean total duration of labour (hour) ± SD	6.22 ± 2.41	8.33 ± 3.56		<0.001*
Oxytocin augmentation of labour, n(%)	48 (43.6)	53 (47.7)	0.91(0.67–1.21)	0.501
Prolonged labour (injection to full cervical dilatation > 12 hours), n(%)	2 (1.8)	8 (7.2)	0.25(0.05–1.15)	0.075
Median Labour pain score [VAS] (IQR)				
At randomization	8 (5 – 10)	7 (5 – 9)		0.968**
At 30 minutes post-randomization	7 (4 – 8)	8 (5 – 10)		0.905**
At 60 minutes post-randomization	8 (6 – 9)	8 (6 – 10)		0.999**
At 120 minutes post-randomization	8 (6 – 10)	8 (6 – 10)		0.999**
Mode of delivery				
Vaginal delivery (including instrumental)	110	111		0.497****
Caesarean section	11	8		
Mean estimated blood loss (ml) ± SD	346.6 ± 86.3	344.8 ± 84.8		0.854*
Neonatal outcome				
Mean birth weight (kg) ± SD	2.62 ± 1.2	2.64 ± 1.2		0.901*
Apgar scores < 7 at 1st minute, n(%)	4 (3.6)	2 (1.8)		0.683***
Apgar scores < 7 at 5th minute, n(%)	1 (0.9)	0 (0)		0.498***
NICU admission, n(%)	6 (5.5)	5 (4.5)		0.757****
Side effects				
Headache, n(%)	2 (1.8)	0 (0)		0.498***
Nausea, n(%)	3 (2.7)	4 (3.6)		1.000***

* Derived from independent sample “t” test

** Derived from Mann-Whitney U test

*** Derived from Fisher's exact test

**** Derived from Chi-square test

SD=standard deviation, IQR=interquartile range, VAS=visual analogue scale

Although they were not statistically significant, the incidence of oxytocin augmentation of labour (p = 0.501) and prolonged labour (p = 0.075) were lower in the drotaverine group when compared to placebo group.

**Figure 2:**
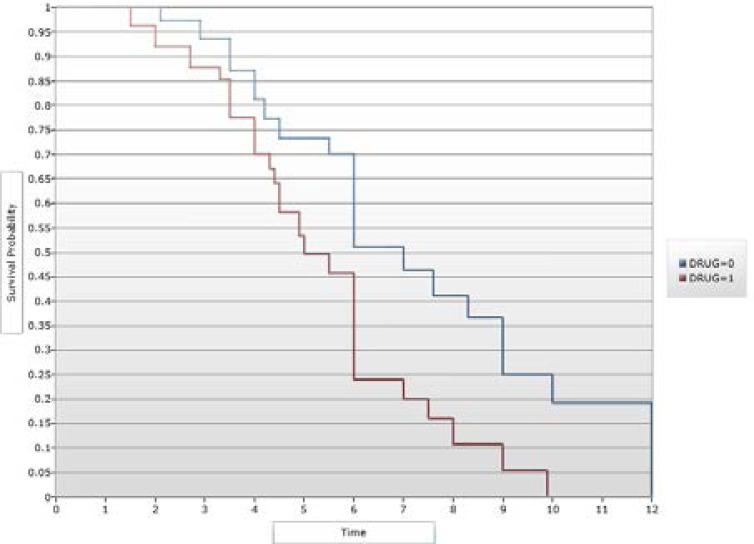
Kaplan-Meier curves showing the probability of faster delivery among women treated with Drotaverine compared with placebo (50% of women reached full cervical dilatation within 5 hours of labour in the drotaverine group compared to 7 hours in the placebo group). Key: Drug-0 depicts the placebo group while drug-1 represents the Drotaverine group.

There were no difference in the labour pain scores at randomization (p = 0.968), 30 minutes (p = 0.905), 60 minutes (p = 0.999) and 120 minutes (p = 0.999) after randomization. The mode of delivery (p = 0.497) and estimated blood loss (p = 0.854) did not differ between the 2 groups. Neonatal adverse events, such as Apgar scores < 7 at 1 and 5 minutes and rate of admission to the neonatal intensive care unit did not differ significantly between the study groups. There was no significant difference between the 2 groups in maternal adverse events: headache was reported for 2 women (1.8%) in the drotaverine group and none for placebo group, whereas nausea was reported for 3 women (2.7%) and 4 women (3.6%) in the drotaverine and placebo groups rspectively. Kaplan-Meier survival analysis is shown in figure 2. The participants who received drotaverine were more likely to achieve faster delivery when compared with those who were administered the placebo (log rank test, P < 0.001).

## Discussion

This study was conducted in the background of active management of labour to assess the effect of drotaverine in shortening the duration of active phase of labour in primigravidas. The baseline variables of the participants in the 2 study groups were similar. Some of these baseline variables can directly or indirectly affect the progress and duration of labour. Therefore, the similarities of baseline variables in both study arms showed that they were unlikely to affect the findings of this study.

This study shows that the rate of cervical dilatation was faster in the drotaverine group when compared to the placebo arm. This finding was similar with the results from previous studies done in which a faster cervical dilation rate was obtained when compared with the placebo.^2^,^14^–^17^ From this study also it can be deduced that a single dose of drotaverine was able to achieve the desired effect when compared with other studies, in which multiple doses of the drug were administered 2 hours apart if labour was not progressing. Ibrahim et al used a standard intrapartum protocol as in the index study but administered multiple doses of the drug intravenously up to 2 repeat doses.^2^ This shows that the desired effect of drotaverine in labour can be achieved with intravenous administration of a single dose of the drug because its half-life is 7 – 12 hours.^7^

The duration of active phase of first stage of labour was significantly shorter in the drotaverine group when compared to the placebo group. However, there were no difference in the duration of second and third stages of labour in the 2 groups. When all the stages of labour were taken into account, the total duration of labour was significantly shorter among participants that received drotaverine when compared to those administered with the placebo. Although not statistically significant, the risk of prolonged labour was lower in the drotaverine group when compared with those in the placebo arm of the study. This finding was similar with the results of other studies.^2^,^8^,^14^–^17^ These findings laid credence to the mechanism of action of drotaverine in accelerating cervical dilatation, its effect being primarily on the cervix and does not affect contractility of the myometrium despite having smooth muscle relaxing effect.

In this study, the rate of oxytocin augmentation did not differ in both study arms. This is probably because drotaverine does not enhance myometrial contractility but rather inhibits type IV phosphodiesterase which is predominantly high in the myometrium during the third trimester and near term.^18^ This process leads to smooth muscle relaxation and cervical dilatation. The oxytocin augmentation rate for this study is similar to that observed by Ibrahim et al.^2^

The caesarean section rate was comparable for both arms of the study. Participants who had caesarean section were 11 and 8 in the drotaverine and placebo arms respectively. The caesarean deliveries observed in both arms were mainly due to cephalopelvic disproportion unlike in the study by Ibrahim, et al2 in which fetal distress was the commonest indication. In this study, the duration of active phase of labour was the primary outcome. Therefore, participants who delivered by caesarean were excluded from data analysis.

In our maternity unit, epidural analgesia in labour is not routine for every woman in labour. Analgesia in labour is achieved with the use of pentazocine or tramadol and none of the study participants received epidural analgesia in labour. This study assessed the beneficial effect of drotaverine in relieving pain in labour. The finding of this study showed that the labour pain scores did not differ in the 2 groups. This is similar to the finding of a study done by Singh et al. that reported no effect of drotaverine on pain relief.^19^ However, it differs from the study by Ibrahim et al. that showed a beneficial effect of drotaverine in relieving labor pains.^2^ This difference is probably because multiple doses of drotaverine were administered unlike in our study where the participants received a single dose of the drug. The repeat doses of the drug may have increased the serum level of the drug. Hence, potentiating its analgesic effect. The other studies reviewed did not assess pain, to determine the analgesic property of drotaverine.^8^,^14^–^16^

Maternal side effects are mild and rare. It usually occurs when the drug is given rapidly during intravenous administration.^2^ In this study, 2 participants had headache in the drotaverine arm and 3 experienced nausea, while in the placebo arm, 4 participants experienced nausea only. Nausea and headache are known side effect of drotaverine and as such these findings from the drotaverine arm of the study is not surprising. Since these findings were not statistically significant, it can be deduced that the side effect profile of drotaverine is safe. Ibrahim et al reported palpitations, hypotension and tachycardia, in addition to nausea and headache in their study.^2^ Palpitations, hypotension and tachycardia were not observed in our study participants. Drotaverine does not cross the placenta and as such has no significant fetal adverse effects.^2^ Comparison of Apgar scores at 1 minute and 5 minutes and neonatal intensive unit admissions were similar for both arms of the study. Ibrahim et al reported similar findings in their studies.^2^ Other studies reviewed did not state the findings of their neonatal outcome or maternal side effects in their reports. More studies are needed to assess the maternal and perinatal side effects of drotaverine.

The study design of a multicenter double-blind placebo-controlled trial is the major strength of this study. In addition, this study was carried out in the background of active management of labour protocol. Besides these strengths, the present study had a limitation. Maternal satisfaction was not assessed in this study.

## Conclusion

Drotaverine is an effective and safe drug for shortening duration of active phase of first stage of labour in primigravidas with term pregnancy.
